# Mass spectrometry imaging spatially identifies complex-type *N*-glycans as putative cartilage degradation markers in human knee osteoarthritis tissue

**DOI:** 10.1007/s00216-022-04289-9

**Published:** 2022-09-20

**Authors:** Yea-Rin Lee, Matthew T. Briggs, Clifford Young, Mark R. Condina, Julia S. Kuliwaba, Paul H. Anderson, Peter Hoffmann

**Affiliations:** 1 Clinical and Health Sciences, Health and Biomedical Innovation, University of South Australia, Adelaide, South Australia Australia; 2 Clinical and Health Sciences, University of South Australia, Adelaide, South Australia 5000 Australia; 3 Discipline of Orthopedics and Trauma, Adelaide Medical School, The University of Adelaide, Adelaide, South Australia Australia

**Keywords:** Knee osteoarthritis, Formalin-fixed paraffin-embedded cartilage tissue, *N*-Glycan, Matrix-assisted laser desorption/ionization mass spectrometry imaging, Liquid chromatography–electrospray ionization–tandem mass spectrometry

## Abstract

*N*-Glycan alterations contribute to the pathophysiology and progression of various diseases. However, the involvement of *N*-glycans in knee osteoarthritis (KOA) progression at the tissue level, especially within articular cartilage, is still poorly understood. Thus, the aim of this study was to spatially map and identify KOA-specific *N*-glycans from formalin-fixed paraffin-embedded (FFPE) osteochondral tissue of the tibial plateau relative to cadaveric control (CTL) tissues. Human FFPE osteochondral tissues from end-stage KOA patients (*n*=3) and CTL individuals (*n*=3), aged >55 years old, were analyzed by matrix‐assisted laser desorption/ionization mass spectrometry imaging (MALDI‐MSI) and liquid chromatography–tandem mass spectrometry (LC-MS/MS). Overall, it was revealed that 22 *N*-glycans were found in the cartilage region of KOA and CTL tissue. Of those, 15 *N*-glycans were more prominent in KOA cartilage than CTL cartilage. We then compared sub-regions of KOA and CTL tissues based on the Osteoarthritis Research Society International (OARSI) histopathological grade (1 to 6), where 1 is an intact cartilage surface and 6 is cartilage surface deformation. Interestingly, three specific complex-type *N*-glycans, (Hex)_4_(HexNAc)_3_, (Hex)_4_(HexNAc)_4_, and (Hex)_5_(HexNAc)_4_, were found to be localized to the superficial fibrillated zone of degraded cartilage (KOA OARSI 2.5-4), compared to adjacent cartilage with less degradation (KOA OARSI 1-2) or relatively healthy cartilage (CTL OARSI 1-2). Our results demonstrate that *N*-glycans specific to degraded cartilage in KOA patients have been identified at the tissue level for the first time. The presence of these *N*-glycans could further be evaluated as potential diagnostic and prognostic biomarkers.

**Supplementary Information:**

The online version contains supplementary material available at 10.1007/s00216-022-04289-9.

## Introduction

Knee osteoarthritis (KOA) is the most common form of joint disease, characterized by the degradation of the articular cartilage. KOA is frequently recognized as a chronic disease that results in disability from performing daily repetitive activities [1]. Chondrocytes are the only type of cells residing in articular cartilage and are responsible for the synthesis and degradation of the extracellular matrix (ECM). The ECM is mainly comprised of collagen fibers, proteoglycans, and glycoproteins. Although KOA is a disorder of the whole joint, the progressive destruction of cartilage ECM via a disequilibrium between anabolism and catabolism is considered its hallmark [2].

Glycans can be classified into two main groups; those attached to asparagine residues (i.e., *N*-glycans) and those attached to serine or threonine residues (i.e., *O*-glycans). However, the relationship between structure and function is much better understood for *N*-glycosylation as *N-*glycans regulate cell adhesion, immune modulation, cell-matrix interactions, and cell proliferation [3]. It has been well documented that *N*-glycosylation changes contribute to pancreatic, ovarian, gastric, colon, and breast cancers [4, 5]. Therefore, it is not surprising that the majority of clinical cancer biomarkers are glycoproteins, such as alpha-fetoprotein for liver cancer, cancer antigen 125 for ovarian cancer, carcinoembryonic antigen for colon cancer, and prostate-specific antigen for prostate cancer [6–9].

In terms of KOA, the pathogenesis of the disease is still poorly understood and the significance of glycan changes at the tissue level has rarely been investigated. However, Matsuhashi et al. [10] first observed *N-*glycan alterations in the cartilage of a rabbit model of surgically induced osteoarthritis (OA), demonstrating there are overall changes in both sialylated and fucosylated *N-*glycans. Later, Urita et al. [11] identified changes in oligomannose-type *N*-glycans in both human and mouse OA cartilage. However, both studies did not obtain spatial information for these particular *N*-glycans. The spatial distribution of different *N*-glycan species between patient samples (or within different tissue types) can be compared with tissue morphology and thereby provide a better understanding of the biological processes occurring within a patient.

With the constant improvements in mass spectrometry imaging (MSI), the use of matrix-assisted laser desorption/ionization (MALDI)–MSI has now been routinely utilized for spatial profiling and identification of *N*-glycans directly from tissue sections without the complications of labeling or staining techniques [12, 13]. The typical approach involves the deposition of the PNGase F enzyme across the surface, to cleave *N*-glycans after which a matrix, such as α-cyano-4-hydroxycinnamic acid (CHCA), is applied for direct detection in-situ by MALDI-MSI [14, 15]. Thus, *N*-glycan MALDI-MSI can be used to correlate morphological changes with molecular information.

Although MALDI-MSI can provide valuable spatial information, the characterization of molecular structural information is limited due to the complexity of the tissue sample limiting the ability to detect all *N*-glycan forms. As such, complementary methods that adopt chromatographic separation coupled with MS improve the sensitivity to detect more *N*-glycans. To overcome this caveat, our group has developed a tissue-based workflow that combines MALDI-MSI and liquid chromatography–tandem mass spectrometry (LC-MS/MS), which uses collision-induced dissociation (CID), to improve the detection of *N*-glycans and yield more fragmentation information to assist in structural characterization of *N*-glycans observed by MALDI-MSI [16]. Thus, the aim of this study was to apply these techniques to spatially map and identify KOA-specific *N*-glycans from formalin-fixed paraffin-embedded (FFPE) osteochondral tissue of the tibial plateau relative to cadaveric control (CTL) tissues.

## Materials and methods

### Materials

Chemicals, consumables, and equipment used for *N-*glycan MALDI-MSI and LC-MS/MS analyses in this study have been previously described [17–20].

### Human tibial plateau tissue specimens

Tibial plateau specimens were collected at the time of surgery from patients undergoing total knee arthroplasty for advanced KOA (*n*=3; aged 73–81 years). The specimens were collected with informed written consent and with approval from the Human Research Ethics Committee at the Royal Adelaide Hospital, in accordance with the Declaration of Helsinki 1975.

Inclusion criterion for KOA tissue was any patient aged over 55 years having an elective total knee replacement for severe symptomatic advanced stage KOA. CTL tibial plateau specimens were obtained from cadavers (*n*=3; aged 59–80 years) with no history of bone or joint disease. Both KOA patients and CTL individuals’ age, gender, and body mass index (BMI) were recorded separately (see Supplementary Table 1).

For both KOA and CTL tibial plateaus, an osteochondral tissue comprising cartilage-subchondral bone (10 × 10 × 5 mm) was cut in the sagittal plane from the medial condyle (a region typically containing more degraded cartilage [21]) using a low-speed diamond wheel saw (Model 660, South Bay Technology, San Clemente, CA, USA). Then, they were further processed in 10% buffered formalin for fixation and 10% tri-ethylenediaminetetraacetic acid (EDTA; pH 8.0) for gentle decalcification and embedded in paraffin wax according to routine histopathology methods [22].

### Tissue sectioning for MALDI-MSI and LC-MS/MS

**Fig. 1:**
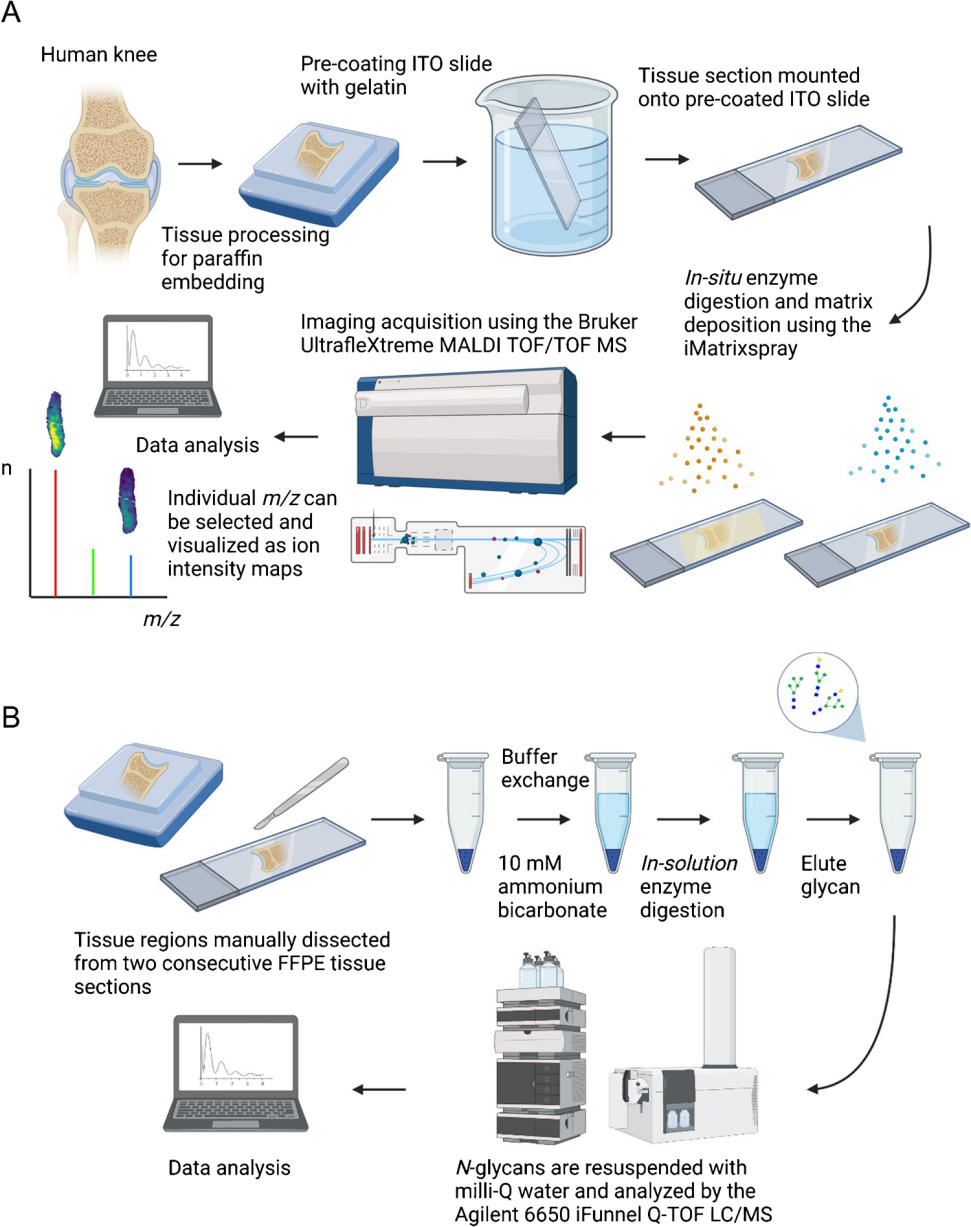
Overview of *in-house* developed **A** MALDI-MSI and **B** LC-ESI-MS/MS workflows for spatial mapping and structural characterization of *N-*glycans released from tibial osteochondral FFPE tissues from KOA patients (*n*=3) and CTL individuals (*n*=3) (created with biorender.com)

FFPE osteochondral tissue blocks were sectioned at 6 µm using a Leica RM2255 fully automated rotary microtome (Nussloch, Germany) and mounted onto gelatin pre-coated indium tin oxide (ITO) slides (Bruker Daltonics, Bremen, Germany) for MALDI-MSI, and polyethylene naphthalate (PEN) membrane slides (Thermo Fisher Scientific, Waltham, MA, USA) for LC-MS/MS, as previously described [16, 19] (see Fig. 1). These slides were left to dry at 37°C in an oven overnight before analysis.

### *N*-glycan MALDI-MSI of FFPE osteochondral tissue sections

#### Tissue antigen retrieval

Tissue sections were heated at 60°C for 1 h on a heat block and cooled to room temperature before deparaffinization. Tissue sections were then washed with 100% (v/v) xylene (2 × 5 min) to remove paraffin and dehydrated with ethanol (2 × 2 min). After that, tissue sections were rehydrated in 10 mM NH_4_HCO_3_ (2 × 5 min), followed by overnight incubation in 10 mM citric acid (pH 6.0) buffer at 37°C in a humid chamber. Tissue sections were placed in a 700W microwave oven and heated for 1 min. The buffer was cooled to room temperature, and the slides were dried at room temperature for 10 min. Finally, tissue sections were immersed in 10 mM NH_4_HCO_3_ (2 × 1 min) and dried at room temperature.

#### In-situ PNGase F digestion and matrix deposition

PNGase F (20 µL) was diluted with 10 mM NH_4_HCO_3_ (2 mL) which was deposited directly onto the tissue sections using the iMatrixSpray instrument (Tardo Gmbh, Subingen, Switzerland) as previously described [19]: 6 mm height, 1 mm line distance, 160 mm/s speed, 1 µL/cm^2^ (9.8 units/cm^2^) density, 30 cycles, 15 s delay, and 80 × 30 mm dimensions. Following the spray, tissue sections were incubated in a sealed container for 2 h at 37°C in a humid chamber with potassium sulfate (53.33 g of potassium sulfate with 18.19 g of milli-Q water to achieve 97% humidity). Peptide calibration standard II (Bruker Daltonics, Bremen, Germany) was manually spotted (1 µL) onto the tissue to calibrate the MALDI-time-of-flight (TOF)-MS instrument. Slides were marked at the edges with water-based whiteout, and tissue sections were scanned at 4800 dpi using a CanoScan 5600 F (Canon, Mississauga, Ontario, Canada) scanner for MALDI-TOF/TOF-MS instrument teaching purposes. Subsequently, CHCA (7 mg/mL in 50% acetonitrile (ACN)/0.2% (v/v) trifluoroacetic acid (TFA)) matrix was sprayed onto tissue sections using the iMatrixSpray instrument using the same settings as PNGase F deposition.

#### MALDI-TOF/TOF-MSI acquisition

MALDI-MSI data were acquired using an ultrafleXtreme MALDI-TOF/TOF-MS (Bruker Daltonics, Bremen, Germany) controlled by flexControl (version 3.4, Bruker Daltonics, Bremen, Germany) and flexImaging (version 4.0, Bruker Daltonics, Bremen, Germany) in positive reflectron mode. Instrument-specific settings were as follows: *m/z* 800−4500 range, 700 Da matrix suppression, 2 kHz laser repetition rate, 5 GS/s 75% laser power, and 2698V detector gain. A laser diameter (2_small) was used with a random walk within a 100 μm raster width. The MALDI-TOF/TOF-MS instrument was externally calibrated using the peptide calibration standard spotted earlier. Additionally, FFPE egg white was used as a quality control to assess if the sample preparation is consistent between individual MALDI-MSI runs (data not shown) [23].

#### Histological staining

Following MALDI-MSI data acquisition, tissue sections were washed in 70% ethanol to remove the matrix and stained with Fast Green solution (Sigma-Aldrich, Dorset, UK) for 20 min, followed by Safranin O solution (Sigma-Aldrich, Dorset, UK) for 5 min. Digital images were acquired with the NanoZoomer (Hamamatsu, Photonics, Japan) after dehydration steps for histological analysis to link MS images with histological information. Each sample was histopathologically graded across the whole tissue section using Osteoarthritis Research Society International (OARSI) grading system by two assessors, blinded to the sample grouping. Then, two cartilage regions, based on OARSI scores between either 1-2 (KOA OARSI 1-2) or 2.5-4 (KOA OARSI 2.5-4), were identified for each KOA patient, representing an area of early and moderate KOA, respectively. For CTLs, cartilage regions with OARSI scores of 1-2 (CTL OARSI 1-2) were selected for comparison.

#### Data processing and spectral evaluation

MALDI-MSI data were analyzed using flexImaging (version 4.0, Bruker Daltonics, Billerica, MA, USA) and SCiLS Lab software package (version 2016b, SCiLS, Bruker Daltonics, Billerica, MA, USA) to generate ion intensity maps to visualize region-specific *N*-glycans. Raw data were loaded and pre-processed by TopHat baseline subtraction, using default settings, and normalization to total ion count (TIC). Ion intensity maps were generated by manually selecting the peaks of interest with weak denoising and automatic hotspot removal. Selected *m/z* values ± 0.3 Da were searched against the UniCarb database (https://unicarb-db.expasy.org/) *via* GlycoMod (http://web.expasy.org/glycomod/) to identify their monosaccharide composition and putative structure. Only *N*-glycans found on the GlyConnect database were selected. These putative *N*-glycan structures were then created using Glycoworkbench (version 2.1) [24]. The *N*-glycan compositions were confirmed by combinations of accurate *m/z*, LC-ESI-MS/MS CID fragmentation patterns, and previously published papers.

For statistical comparisons, mean intensity values ± standard deviations were calculated for each *N*-glycan using SCiLS Lab software and exported as an Excel file. These values were then tested for normal distribution based on a Shapiro-Wilk test using the GraphPad Prism 8 software (GraphPad Software, Inc., San Diego, CA, USA). If data was normally distributed, an ANOVA test was used to determine the *p*-value. Otherwise, a Kruskal-Wallis test was performed.

### *N*-glycan structural confirmation by LC-ESI-MS/MS

#### In-solution PNGase F digestion

Consecutive tissue sections were mounted onto PEN membrane slides and processed as previously described [16, 17, 20, 25]. Briefly, tissue sections were heated for 5 min at 60°C, prior to 90 s xylene and 60 s ethanol washes. Two consecutive cartilage tissue sections (20 × 5 mm) were manually micro-dissected using a sterile disposable surgical knife and incubated (2 × 5 min) in 200 µL of 10 mM NH_4_HCO_3_, followed by 45 min with 200 µL of 10 mM citric acid pH 6.0 at 98 °C. Tissues were then washed twice with 200 µL of 10 mM NH_4_HCO_3_ and digested with 2 µL of PNGase F in 40 µL of 25 mM NH_4_HCO_3_ overnight at 37°C.

#### *N*‐glycan release and glycan purification

The released *N*‐glycans were reduced and desalted according to previously described methods, with some modifications [16, 17, 20, 25]. Briefly, after acidification with 100 mM NH_4_COOH at pH 5 (10 µL) for 60 min at room temperature, samples were dried in a vacuum centrifuge and reduced with 1 M NaBH_4_ in 50 mM NaOH (20 µL) at 50 °C for 3 h. The reduction was quenched with 2 µL glacial acetic acid and desalted using cation‐exchange columns comprising 30 µL of AG50W‐X8 cation‐exchange resin (BioRad, Hercules, CA, USA) in methanol pipetted on top of C_18_ ZipTips (Merck Millipore, Rockland, MA, USA). Each column was washed prior to loading the glycan samples with 1 M hydrochloric acid (50 µL × 3) followed by methanol (50 µL × 3) and milli-Q water (50 µL × 3). Residual borate was removed by the addition of methanol (100 µL × 3) and dried in the SpeedVac concentrator without heating. The *N*-glycans were further purified by tips using 50 µL (~2.5 mg) of a carbon slurry (GlycoClean H Cartridges, Prozyme) in methanol manually pipetted onto C_18_ ZipTips (Merck Millipore, Rockland, MA, USA). Again, the columns were washed with ACN containing 0.1% (v/v) TFA (50 µL × 3), followed by milli-Q water containing 0.1% (v/v) TFA (50 µL × 3). Bound glycans were eluted by adding 50% (v/v) ACN containing 0.1% (v/v) TFA (10 µL × 2) and dried in the SpeedVac concentrator without heating.

#### Porous graphic carbon (PGC)-LC-ESI-MS/MS analysis

The released *N*‐glycans were resuspended in milli-Q water (10 µL) and analyzed using a 1290 Infinity II LC System (Agilent Technologies, Palo Alto, CA, USA) coupled to a 6550 iFunnel Q-TOF mass spectrometer (Agilent Technologies). PGC-LC-ESI-MS/MS settings were the same as previously described [20].

#### Data processing

Raw data were processed by the MassHunter Qualitative Analysis software (version B.8.00, Agilent Technologies, Santa Clara, CA, USA). Extracted ion chromatograms (EICs) were generated based on an *in-house N*-glycan library of doubly charged species. Then, observed *N*-glycan structures were generated in GlycoWorkbench software and confirmed by MS/MS CID fragmentation data which involved manual assignment and annotation of B/C/Y/Z-type fragment ions using GlycoWorkbench software [24, 26].

## Results

### Overall *N*-glycan profiles for CTL and KOA tissue samples

**Fig. 2:**
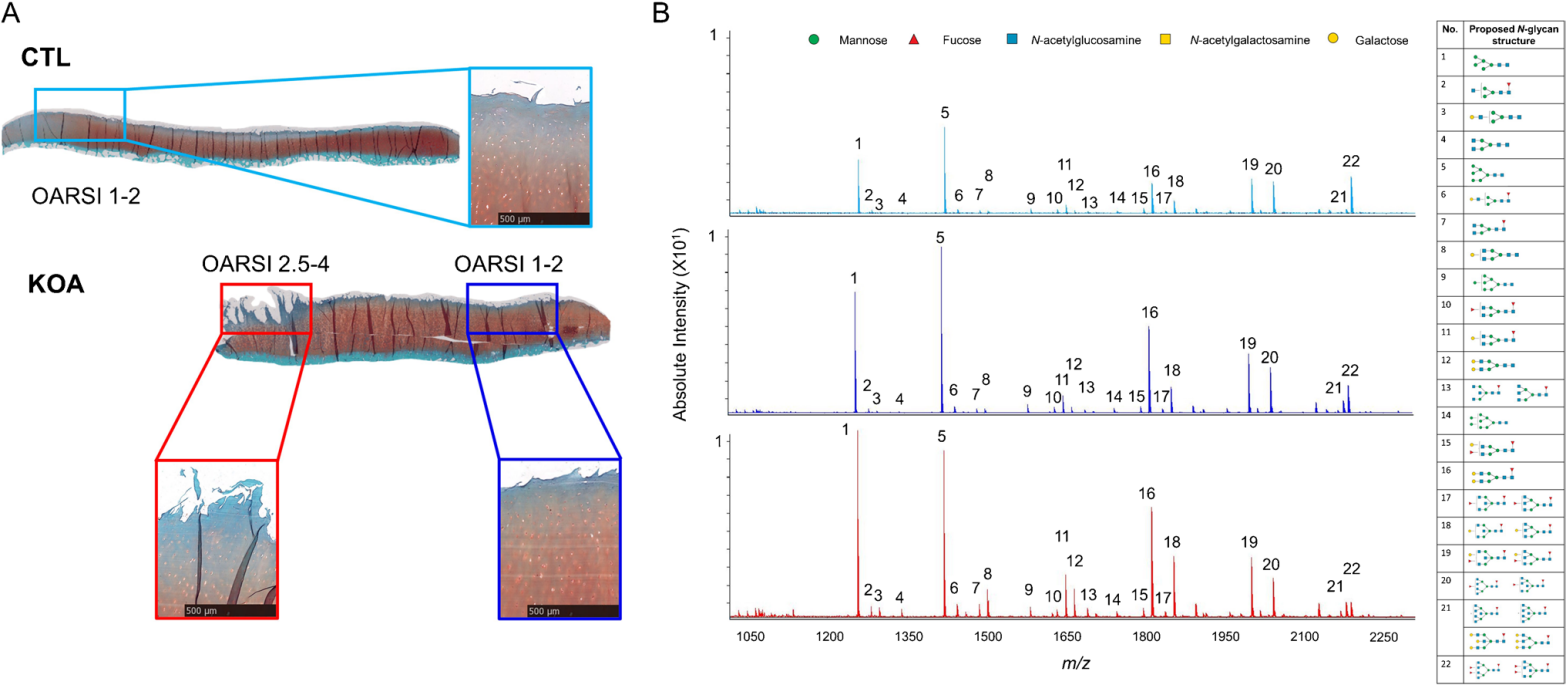
**A** Representative Fast Green/Safranin O stained images of CTL and KOA tissue sections demonstrating superficial fibrillation (OARSI 1-2) and discontinuity and vertical fissures (OARSI 2.5-4) of the articular cartilage superficial layer. **B** Overall comparison of summed spectra for CTL individuals (*n*=3) and KOA patients (*n*=3) analyzed by MALDI-MSI with a *m/z* range of 800–4500 in positive reflectron mode. Raw spectra were processed by performing TopHat baseline subtraction and normalization to TIC in SCiLS Lab software

In order to compare the summed spectra of *N*-glycans between CTL individuals (*n*=3) and KOA patients (*n*=3), the OARSI histopathological grades (1–6) were determined for each sample, where 1 is intact cartilage surface and 6 is cartilage surface deformation. This resulted in three sample classifications: CTL with OARSI grades 1–2, KOA with OARSI grades 1–2, and KOA with OARSI grades 2.5–4 (see Fig. 2). Raw spectra were processed by performing TopHat baseline subtraction, using default settings, and normalization to TIC in SCiLS Lab software. In total, 22 *N*-glycans were detected by MALDI-MSI from both CTL individuals and KOA patients, and their compositions and putative structures were determined using the UniCarb database, accessed via GlycoMod (see Supplementary Table 2). Overall, 15 *N*-glycans were more prominent in KOA cartilage than CTL cartilage. Furthermore, when OARSI graded regions were compared, it showed that 12 out of 22 *N*-glycans were more intense in the degraded cartilage (KOA OARSI 2.5-4) compared to adjacent cartilage with less degradation (KOA OARSI 1-2) or relatively healthy cartilage (CTL OARSI 1-2) (see Fig. 2).

### Three *N*-glycans were found to be specific to degraded KOA cartilage

Statistical analysis of the 22 *N*-glycans was conducted, comparing CTL OARSI 1-2, KOA OARSI 1-2, and KOA OARSI 2.5-4, using the mean intensities ± standard deviation calculated and exported from SCiLS Lab software. The statistical significance threshold was set as *p* < 0.05 and the analyses were performed using the GraphPad Prism 8 software.

**Fig. 3:**
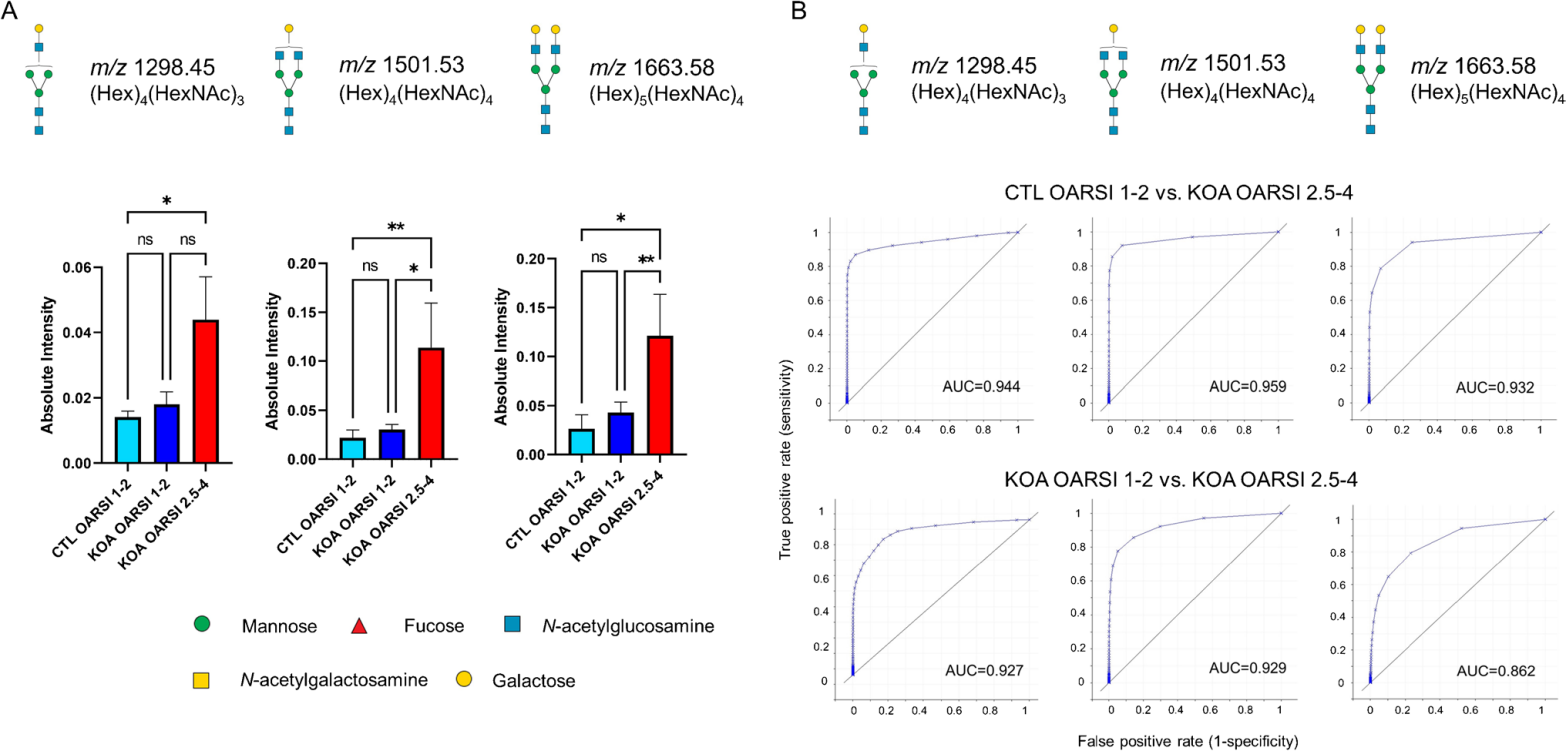
**A** Normalized absolute intensity of three complex-type *N*-glycans, (Hex)_4_(HexNAc)_3_, (Hex)_4_(HexNAc)_4_, and (Hex)_5_(HexNAc)_4_, for OARSI graded regions, CTL OARSI 1-2, KOA OARSI 1-2, and KOA OARSI 2.5-4. The mean intensities ± standard deviation were exported from SCiLS Lab software and the statistical analyses were performed using the GraphPad Prism 8 software. *p value <0.05, **p value <0.01, and ns: non-significant. **B** ROC plots of the same three *N*-glycans for CTL OARSI 1-2 and KOA OARSI 2.5-4, and KOA OARSI 1-2 and KOA OARSI 2.5-4

Complex-type *N*-glycans, (Hex)_4_(HexNAc)_3_, (Hex)_4_(HexNAc)_4_, and (Hex)_5_(HexNAc)_4_, significantly increased in intensity from CTL OARSI 1-2 to KOA OARSI 2.5-4 (*p*=0.0219, *p*=0.0094 and *p*=0.0069; respectively). Additionally, (Hex)_4_(HexNAc)_4_ and (Hex)_5_(HexNAc)_4_ also significantly increased between KOA OARSI 1-2 and KOA OARSI 2.5-4 (*p*=0.0143 and *p*=0.0164; respectively), as represented in Fig. 3. The statistical analyses for the remaining *N-*glycans are shown in Supplementary Fig. 1.

Receiver operating curve (ROC) analysis and determination of the area under the ROC curve (AUC) were also carried out to determine the specificity and sensitivity of (Hex)_4_(HexNAc)_3_, (Hex)_4_(HexNAc)_4_, and (Hex)_5_(HexNAc)_4_ between CTL OARSI 1-2 and KOA OARSI 2.5-4, and KOA OARSI 1-2 and KOA OARSI 2.5-4 (see Fig. 3). These complex-type *N*-glycans demonstrated high AUC values, ranging between 0.862 and 0.959, confirming their potential power as highly specific markers for KOA OARSI 2.5-4 relative to both CTL and KOA samples with OARSI 1-2 (see Fig. 3). The comparison for CTL OARSI 1-2 and KOA OARSI 1-2 is shown in Supplementary Fig. 2.

**Fig. 4:**
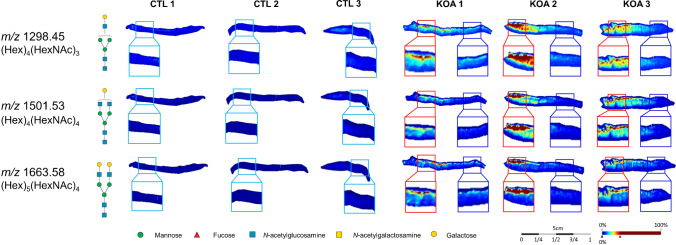
*N-*glycan MALDI-MSI of FFPE cartilage tissue from CTL individuals (*n*=3) and KOA patients (*n*=3). Ion intensity maps were generated and visualized for *m/z* 1298.45, 1501.53, and 1663.58 ± 0.3 Da using SCiLS Lab software. These three complex-type *N*-glycans, (Hex)_4_(HexNAc)_3_, (Hex)_4_(HexNAc)_4_, and (Hex)_5_(HexNAc)_4_, were localized to the upper superficial surface in KOA OARSI 2.5-4 relative to CTL OARSI 1-2 and KOA OARSI 1-2

Ion intensity maps were then generated for *m/z* 1298.45, 1501.53, and 1663.58 ± 0.3 Da using SCiLS Lab software. Interestingly, all three complex-type *N-*glycans, (Hex)_4_(HexNAc)_3_, (Hex)_4_(HexNAc)_4_, and (Hex)_5_(HexNAc)_4_, were predominantly localized in the upper fibrillated surface of the degraded cartilage region (KOA OARSI 2.5-4) compared to adjacent cartilage with less degradation (KOA OARSI 1) or relatively healthy cartilage (CTL OARSI 1-2) (see Fig. 4). Ion intensity maps for the remaining *N*-glycans are shown in Supplementary Fig. 3.

**Fig. 5:**
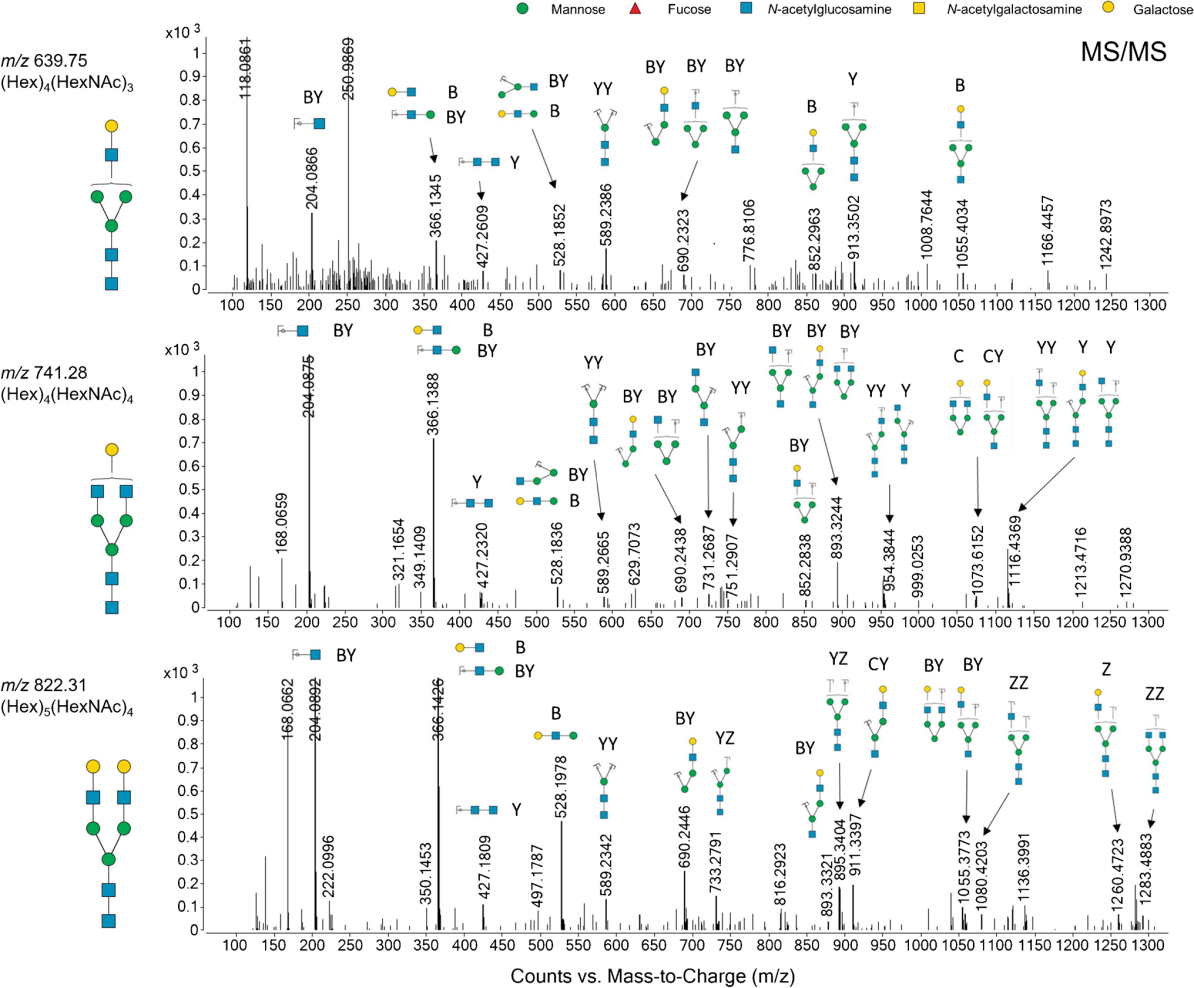
PGC-LC-ESI-MS/MS revealed doubly positive charged *N-*glycan species [M+2H]^2+^ of *m/z* 639.75, 741.28, and 822.31. These doubly charged *N-*glycan species were calculated based on the singly sodiated *N-*glycan species observed from the MALDI-MSI of *m/z* 1298.45, 1501.53, and 1663.58. Theoretical B/C/Y/Z-type ions were generated using GlycoWorkbench software and assigned to ions of the same *m/z* value in the fragment spectra to confirm the structural identity of each *N-*glycan

To confirm the putative structure assigned to these three complex-type *N*-glycans, consecutive tissue sections were digested *in-solution* using PNGase F, desalted, purified, and analyzed by PGC-LC-ESI-MS/MS. All *N*-glycans were identified as doubly charged ions in CID positive mode. Doubly charged species were generated, based on the singly sodiated species [M+Na]^1+^ observed during MALDI-MSI, using the MassHunter Qualitative Analysis software. Each *N-*glycan structure was then manually confirmed for (Hex)_4_(HexNAc)_3_, (Hex)_4_(HexNAc)_4_, and (Hex)_5_(HexNAc)_4_, based on the B/C/Y/Z-type fragment ions observed and matched to theoretical fragment ions generated using GlycoWorkbench software (see Fig. 5). These B/C/Y/Z-type fragment ions originate from different types of glycosidic bond cleavages, with B- and Y-type ions being more common than C- and Z-type ions [27, 28].

## Discussion

KOA pathogenesis is poorly understood due to a lack of insight into the molecular events that occur during KOA cartilage degeneration. *N*-glycosylation has been reported to be involved in the pathogenesis of several diseases [29, 30]. However, the investigation of *N*-glycosylation in OA in general has been limited mainly to serum and cell line studies [10, 11, 31]. To determine which *N*-glycans are specific to degraded cartilage in osteoarthritic joints, we have applied an *in-house* gelatin pre-coating MALDI-MSI protocol, developed for hard-fragile osteochondral tissues [19], to assess *N-*glycan alterations compared between KOA samples and CTL specimens. *N-*glycan abundance and spatial distribution were evaluated within mild to moderate degenerate cartilage regions defined by the OARSI histopathological grade for end-stage KOA patients in comparison to CTL tissue samples.

Although 22 *N-*glycans were commonly detected in the cartilage of both KOA and CTL specimens, 12 out of 22 *N*-glycans were higher in abundance in the degraded cartilage (KOA OARSI 2.5-4) compared to adjacent cartilage with less degradation (KOA OARSI 1-2) or relatively healthy cartilage (CTL OARSI 1-2). This observation suggests that *N*-glycan changes in cartilage are associated with histopathological changes, increasing as a function of disease progression. Furthermore, among 12 *N*-glycans predominantly identified within the KOA OARSI 2.5-4 cartilage, three specific complex-type *N*-glycans, (Hex)_4_(HexNAc)_3_, (Hex)_4_(HexNAc)_4_, and (Hex)_5_(HexNAc)_4_, were predominantly localized to the upper (superficial layer) fibrillated surface of the degraded KOA cartilage region (KOA OARSI 2.5-4), compared to adjacent cartilage with less degradation (KOA OARSI 1-2) or relatively healthy cartilage (CTL OARSI 1-2). The identification of these *N*-glycans is consistent with our previous reports [16, 19].

It has also been reported that (Hex)_4_(HexNAc)_3_ and (Hex)_4_(HexNAc)_4_ were identified in serum at significantly higher levels in OA and rheumatoid arthritis (RA) groups when compared to healthy control serum by MALDI-TOF-MS analysis [32]. Conversely, the relative intensity of (Hex)_5_(HexNAc)_4_ was significantly lower in the OA and RA group [32], which is in contrast to the higher levels of the (Hex)_5_(HexNAc)_4_ in OA cartilage tissue that we have observed (see Fig. 3). Therefore, these findings suggest that fluid samples, such as synovial fluid, may be an accessible sample type for assessing *N*-glycan biomarkers of KOA progression in the future. It is not surprising that KOA synovial fluid samples are still poorly understood at the *N*-glycan level as limited studies have been investigated, and therefore further research is needed.

*N*-glycan alterations in OA chondrocytes have also been previously shown with oligomannosidic structures, as well as non-, mono-, and disialylated complex-type structures, identified as playing an important role in pathogenesis [31]. Toegel et al. [31] further demonstrated that human galectin-3 was dependent on the grade of cartilage degeneration, and glycoprotein asialofetuin-positive cells were observed in significantly higher numbers in areas of severe degeneration. Based on these findings, it can be postulated that galectins could be present in OA cartilage as on-site effectors that can translate the sugar code of cells and matrix into biological functions. Fuehrer et al. [33] also hypothesized that *N*-glycosylation could reflect phenotypic changes in osteoarthritic cells in vitro. Like chondrocytes, fibroblast-like synoviocytes express *N*-glycans that are suited to bind galectins-1 and -3, and these proteins serve as inducers of pro-inflammatory markers, such as interleukin-1β [33]. This data suggests there is a potential role for lectins to selectively manipulate glycoprotein function in OA disease. However, they did not demonstrate any evidence of galectins and *N*-glycan co-expression at the tissue level, which warrants further investigation.

Interestingly, a recent tissue study confirmed the interaction between two glycoproteins, lubricin and cartilage oligomeric matrix protein (COMP), on the cartilage superficial surface by proximity ligation assay (PLA) [34]. The PLA signal from the lubricin-COMP complex was up to 40 µm below the cartilage surface, and particularly intense where the surface layer was diminished [34]. PLA signals were also detected for the lubricin and fibronectin or collagen II pairs. The latter two pairs were only identified at the cartilage surface with no protein interactions apparent deeper into the tissue. This suggests that both fibronectin and collagen II along with COMP may be involved in the adherence of lubricin to the cartilage surface.

Furthermore, the spatial distribution of well-known OA-related proteins and peptides in cartilage has been previously reported by MALDI-MSI. In this work, fibronectin tryptic peptides were more abundant in OA cartilage relative to control tissues [35]. COMP tryptic peptides also showed a similar spatial distribution to fibronectin [35]. MALDI-MSI has also been employed to investigate the distribution of ECM proteins in young, aged, and OA equine cartilage. Tryptic peptides corresponding to COMP showed higher expression in the same samples whether they were young or old, than in OA cartilage, whereas the collectin-43 protein was specific for young cartilage [36]. These findings led to the hypothesis that glycan modifications of those proteins may contribute to chondrocytes undergoing hypertrophy-like changes, leading to KOA pathogenesis. Although the present study showed that the localization of complex-type *N*-glycans in the superficial layer is associated with disease severity, their biological function is unclear as it was not determined what glycoproteins these *N*-glycans were attached to. Therefore, further studies will be required to obtain a deeper understanding of the glycoproteome and its relationship with KOA pathogenesis.

Although the current study has a slightly improved number of KOA patient samples than our previous studies [16, 19], we are still limited by the patient cohort selected for this study. In most cases, we had to discard tibial plateau specimens as minimal amounts of intact cartilage were left from the end-stage KOA patients. In addition to more patient samples, it would be beneficial to also analyze synovial fluid, collected from the same patients to obtain an overview of the glycomic changes within the surrounding regions of KOA. *N*-glycome analysis in synovial fluid from KOA patients may serve as a less invasive way of identifying cartilage degradation and therefore be developed as a novel source of KOA biomarkers. Another caveat is that positive mode CID provides limited structural information compared to negative mode CID which was attempted without success. Although these limitations are evident, the results of this study demonstrate that complex-type *N*-glycans may be promising as potential diagnostic markers or therapeutic targets.

## Conclusions

For the first time, *N*‐glycan profiling of osteochondral tissue has been investigated in the context of mild to moderate degenerative cartilage, based on the OARSI histopathological grading (1 to 6). Interestingly, we discovered that (Hex)_4_(HexNAc)_3_, (Hex)_4_(HexNAc)_4_, and (Hex)_5_(HexNAc)_4_, originating from the same complex-type *N-*glycan family, were prominent on the superficial fibrillated surface of degraded cartilage (KOA OARSI 2.5-4), compared to adjacent cartilage with less degradation (KOA OARSI 1-2) or relatively healthy cartilage (CTL OARSI 1-2). Therefore, these three complex-type *N*-glycans could be potential KOA cartilage degradation markers. However, further investigations are required to validate these observations using a larger patient cohort, as well as the need to understand the significance of complex-type *N*-glycans in cartilage ECM metabolism in relation to KOA progression.

## Supplementary Information

Below is the link to the electronic supplementary material.

Supplementary file1 (DOCX 1518 KB)

## Abbreviations

AUC: Area under the curve
BMI: Body mass index
CHCA: α-Cyano-4-hydroxycinnamic acid
CID: Collision-induced dissociation
COMP: Cartilage oligomeric matrix protein
CTL: Cadaveric control
ECM: Extracellular matrix
EDTA: Ethylenediaminetetraacetic acid
EIC: Extracted ion chromatograms
FFPE: Formalin-fixed paraffin-embedded
ITO: Indium tin oxide
KOA: Knee osteoarthritis
MALDI: Matrix-assisted laser desorption/ionization
MSI: Mass spectrometry imaging
OARSI: Osteoarthritis Research Society International
PEN: Polyethylene naphthalate
PGC-LC-ESI-MS/MS: Porous graphic carbon–liquid chromatography–electrospray ionization–mass spectrometry
PLA: Proximity ligation assay
PNGaseF: Peptide-N-glycosidase F
RA: Rheumatoid arthritis
ROC: Receiver operating characteristics
TIC: Total ion count
TOF: Time of flight

## Declarations

### Source of biological material

Specimens were collected with informed written consent from all patients and with approval from the Human Research Ethics Committee at the Royal Adelaide Hospital, South Australia.

All authors have read and agreed to the published version of the manuscript.
