# Supplementary material for: Mass spectrometry imaging spatially identifies complex-type N-glycans as putative cartilage degradation markers in human knee osteoarthritis tissue
Source: Anal Bioanal Chem. 2022 Sep 20;414(26):7597–607. doi: 10.1007/s00216-022-04289-9 (PMC9587078; doi:10.1007/s00216-022-04289-9)
Supplement: Supplementary file 1 — Supplementary file1 (DOCX 1518 KB) [file 216_2022_4289_MOESM1_ESM.docx]

**Supplementary Data**

**Supplementary Table 1.** Demographic characteristics of KOA patients (n=3) and CTL individuals (n=3), including their age, BMI and OARSI graded regions of interest (ROI).

|  | **KOA 1** | **KOA 2** | **KOA 3** | **CTL 1** | **CTL 2** | **CTL 3** |
| --- | --- | --- | --- | --- | --- | --- |
| Gender | Male | Female | Male | Male | Male | Male |
| Age | 73 | 81 | 75 | 59 | 78 | 80 |
| Body mass index; BMI (kg/m^2^) | 28 | 25.5 | 27.8 | 32 | 22 | 24 |
| OARSI grade (1 to 6) | KOA ROI 1 – 1.5  KOA ROI 2 – 2.5 | KOA ROI 1 – 1.5  KOA ROI 2 – 3.5 | KOA ROI 1 – 1.5  KOA ROI 2 – 4 | CTL ROI 1 – 1 | CTL ROI 1 – 1 | CTL ROI 1 – 1.75 |

**Supplementary Table 2.** List of *N*-glycans detected by MALDI-MSI as singly charged species [M+Na]^1+^ and their structures characterized by LC-ESI-MS/MS as doubly charged species [M+2H]^2+^ *N*-glycans were released and identified from tibial osteochondral FFPE tissues from KOA patients (n=3) and CTL individuals (n=3). Y=Yes, N=No.

| No. | Glycan composition | Proposed *N*-glycan structure | Detected *m/z* [M+Na]^1+^ from MALDI-MSI | | | | Detected *m/z* [M+2H]^2+^ from LC-MS/MS | | |
| --- | --- | --- | --- | --- | --- | --- | --- | --- | --- |
|  | | | | CTL OARSI 1-2 | KOA OARSI 1-2 | KOA OARSI 2.5-4 |  | CTL | KOA |
| 1 | (Hex)_5_(HexNAc)_2_ | 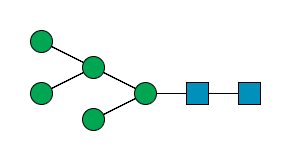 | 1257.42 | Y | Y | Y | 619.23 | Y | Y |
| 2 | (Hex)_3_(HexNAc)_3_(Deoxyhexose)_1_ | 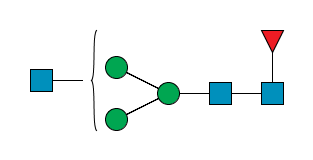 | 1282.45 | Y | Y | Y | 631.75 | Y | Y |
| 3 | (Hex)_4_(HexNAc)_3_ | 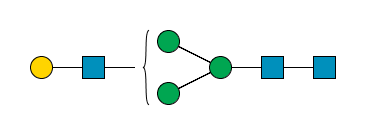 | 1298.45 | Y | Y | Y | 639.75 | Y | Y |
| 4 | (Hex)_3_(HexNAc)_4_ | 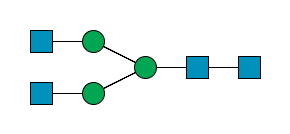 | 1339.48 | Y | Y | Y | 660.26 | Y | Y |
| 5 | (Hex)_6_(HexNAc)_2_ | 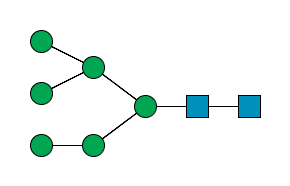 | 1419.48 | Y | Y | Y | 700.26 | Y | Y |
| 6 | (Hex)_4_(HexNAc)_3_(Deoxyhexose)_1_ | **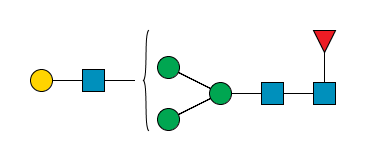** | 1444.51 | Y | Y | Y | 712.77 | Y | Y |
| 7 | (Hex)_3_(HexNAc)_4_(Deoxyhexose)_1_ | 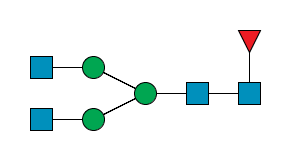 | 1485.53 | Y | Y | Y | 733.29 | Y | Y |
| 8 | (Hex)_4_(HexNAc)_4_ | 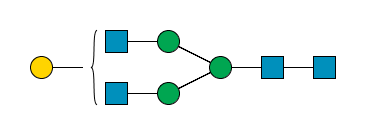 | 1501.53 | Y | Y | Y | 741.28 | Y | Y |
| 9 | (Hex)_7_(HexNAc)_2_ | 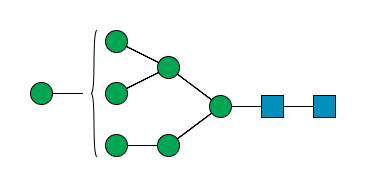 | 1581.53 | Y | Y | Y | 781.28 | Y | Y |
| 10 | (Hex)_3_(HexNAc)_4_(Deoxyhexose)_2_ | 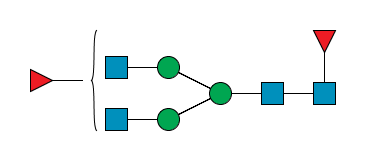 | 1631.59 | Y | Y | Y | 806.32 | Y | Y |
| 11 | (Hex)_4_(HexNAc)_4_(Deoxyhexose)_1_ | 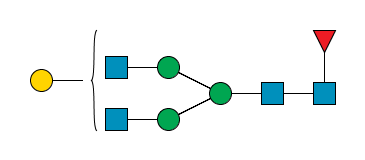 | 1647.59 | Y | Y | Y | 814.31 | Y | Y |
| 12 | (Hex)_5_(HexNAc)_4_ | 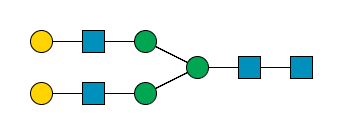 | 1663.58 | Y | Y | Y | 822.31 | Y | Y |
| 13 | (Hex)_3_(HexNAc)_5_(Deoxyhexose)_1_ | 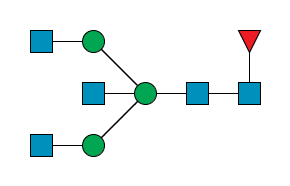  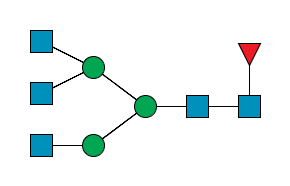 | 1688.61 | Y | Y | Y | 834.83 | Y | Y |
| 14 | (Hex)_8_(HexNAc)_2_ | 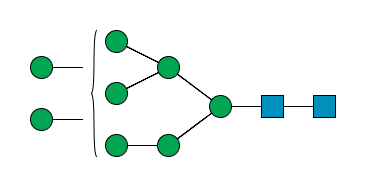 | 1743.58 | Y | Y | Y | 862.31 | Y | Y |
| 15 | (Hex)_4_(HexNAc)_4_(Deoxyhexose)_2_ | 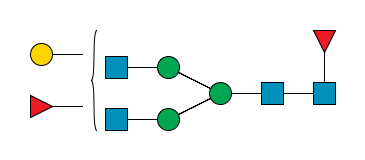 | 1793.64 | Y | Y | Y | 887.34 | Y | Y |
| 16 | (Hex)_5_(HexNAc)_4_(Deoxyhexose)_1_ | 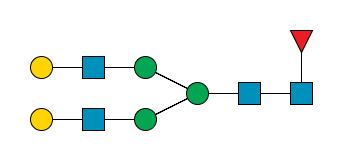 | 1809.64 | Y | Y | Y | 895.33 | Y | Y |
| 17 | (Hex)_3_(HexNAc)_5_(Deoxyhexose)_2_ | 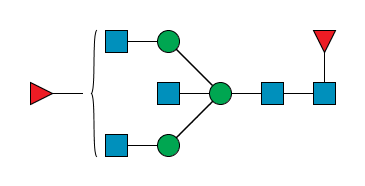  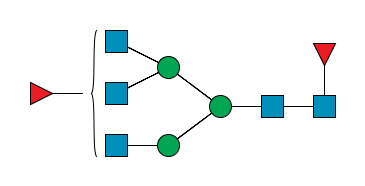 | 1834.67 | Y | Y | Y | 907.86 | Y | Y |
| 18 | (Hex)_4_(HexNAc)_5_(Deoxyhexose)_1_ | 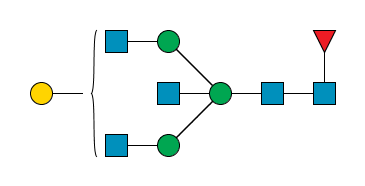  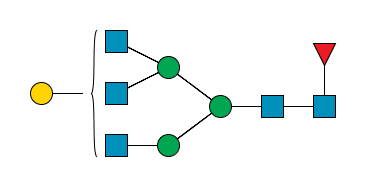 | 1850.67 | Y | Y | Y | 915.85 | Y | Y |
| 19 | (Hex)_4_(HexNAc)_5_(Deoxyhexose)_2_ | 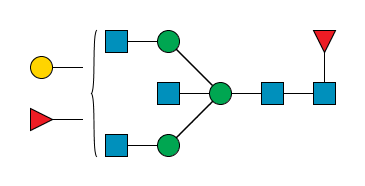  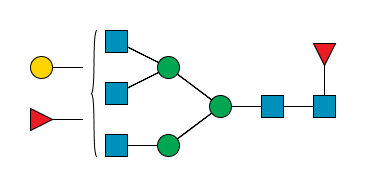 | 1996.72 | Y | Y | Y | 988.88 | Y | Y |
| 20 | (Hex)_3_(HexNAc)_6_(Deoxyhexose)_2_ | 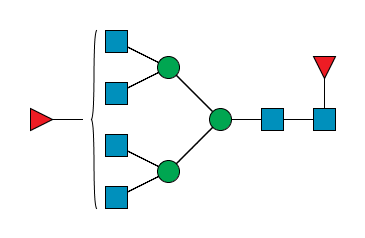  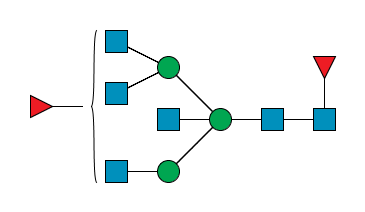 | 2037.75 | Y | Y | Y | 1009.40 | Y | Y |
| 21 | (Hex)_3_(HexNAc)_7_(Deoxyhexose)_1_(Sulph)_1_  **_OR_**  (Hex)_3_(HexNAc)_7_(Deoxyhexose)_1_ (Phos)_1_  **_OR_**  (Hex)_6_(HexNAc)_5_(Deoxyhexose)_1_ | 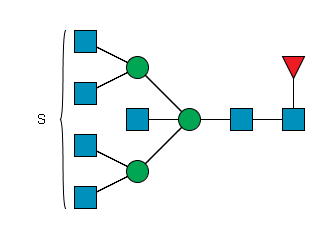  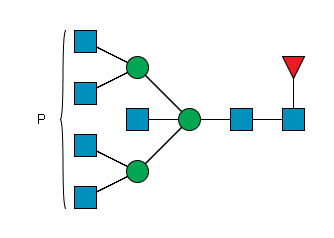  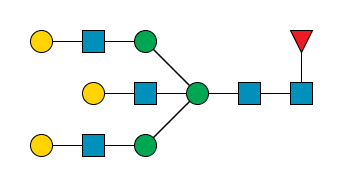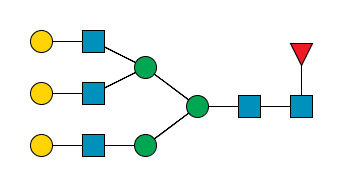 | 2174.77 | Y | Y | Y | 1077.91 | Y | Y |
| 22 | (Hex)_3_(HexNAc)_6_(Deoxyhexose)_3_ | 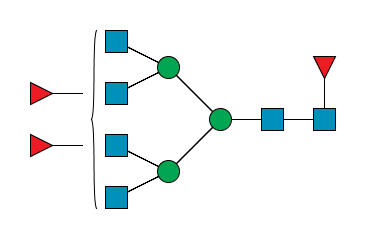  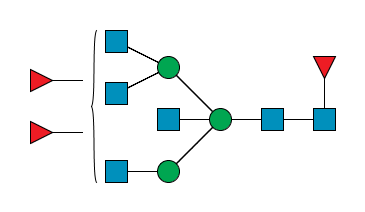 | 2183.81 | Y | Y | Y | 1082.42 | Y | Y |


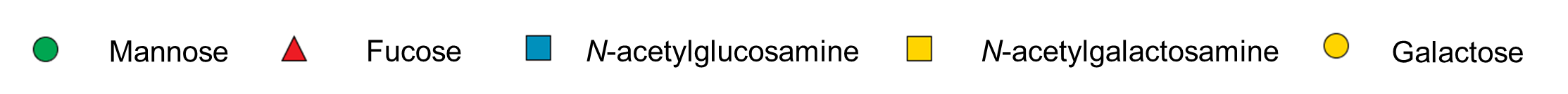


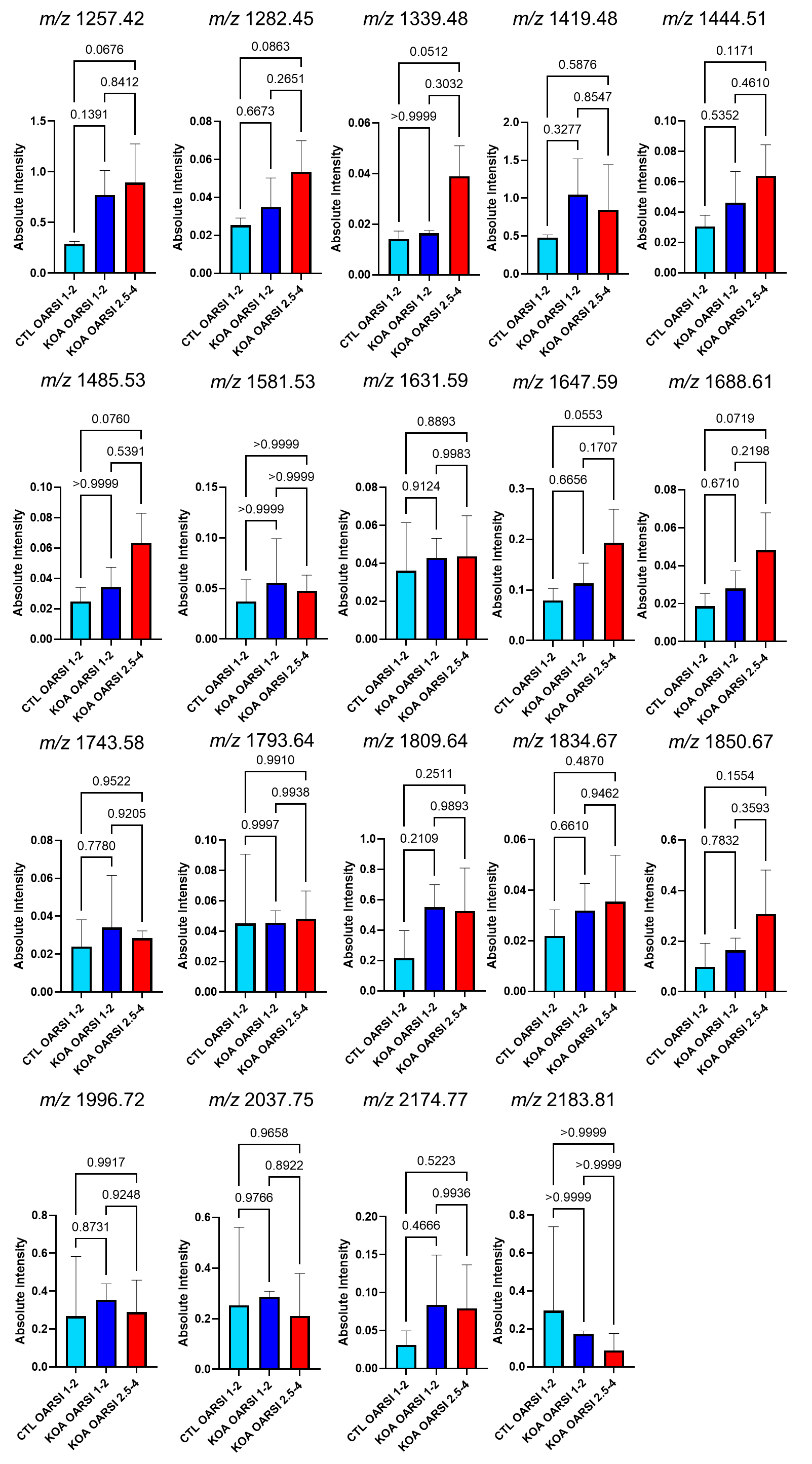


**Supplementary Figure 1.** Statistical analysis of individual *N*-glycans detected by MALDI-MSI were conducted between CTL OARSI 1-2, KOA OARSI 1-2, and KOA OARSI 2.5-4 using the mean intensities ± standard deviation exported from SCiLS Lab software. *N*-glycan intensity data were either normally or non-normally distributed (Shapiro-Wilk tests). The group differences were calculated using ANOVA tests. The statistical significance was chosen as p < 0.05. The analyses were performed using the GraphPad Prism 8 software.


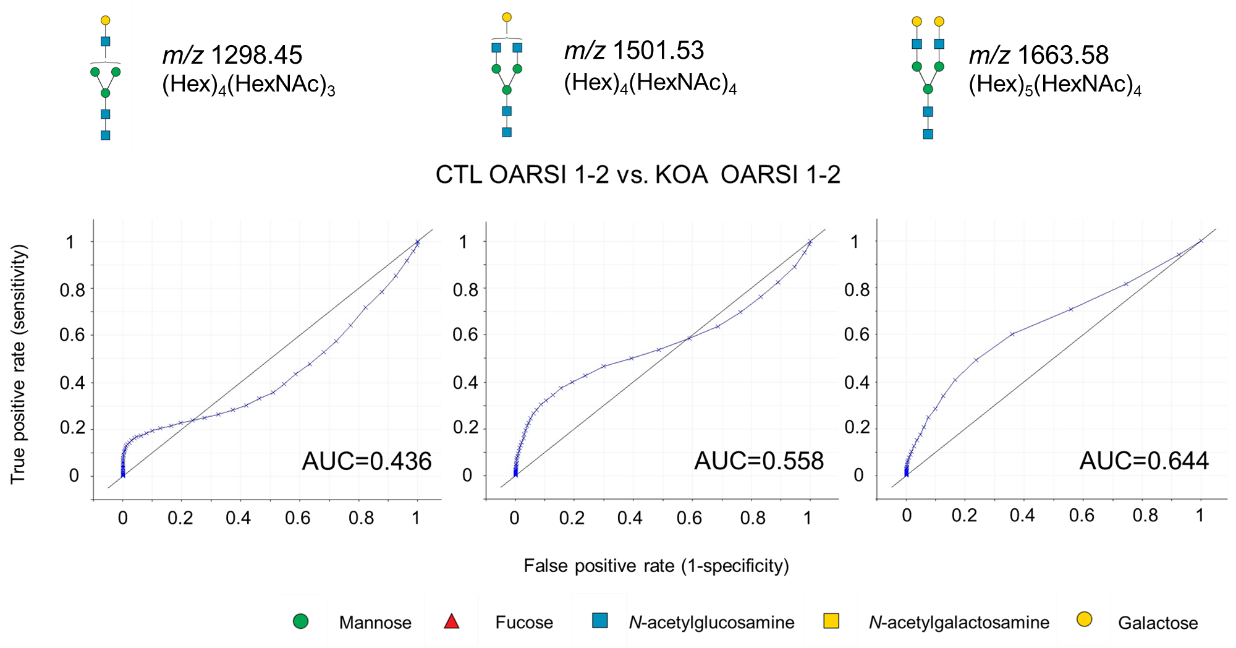


**Supplementary Figure 2.** ROC plots for the *N*-glycans of *m/z* 1298.45, 1501.53, and 1663.58 between CTL OARSI 1-2 and KOA OARSI 1-2 using SCiLS Lab software.


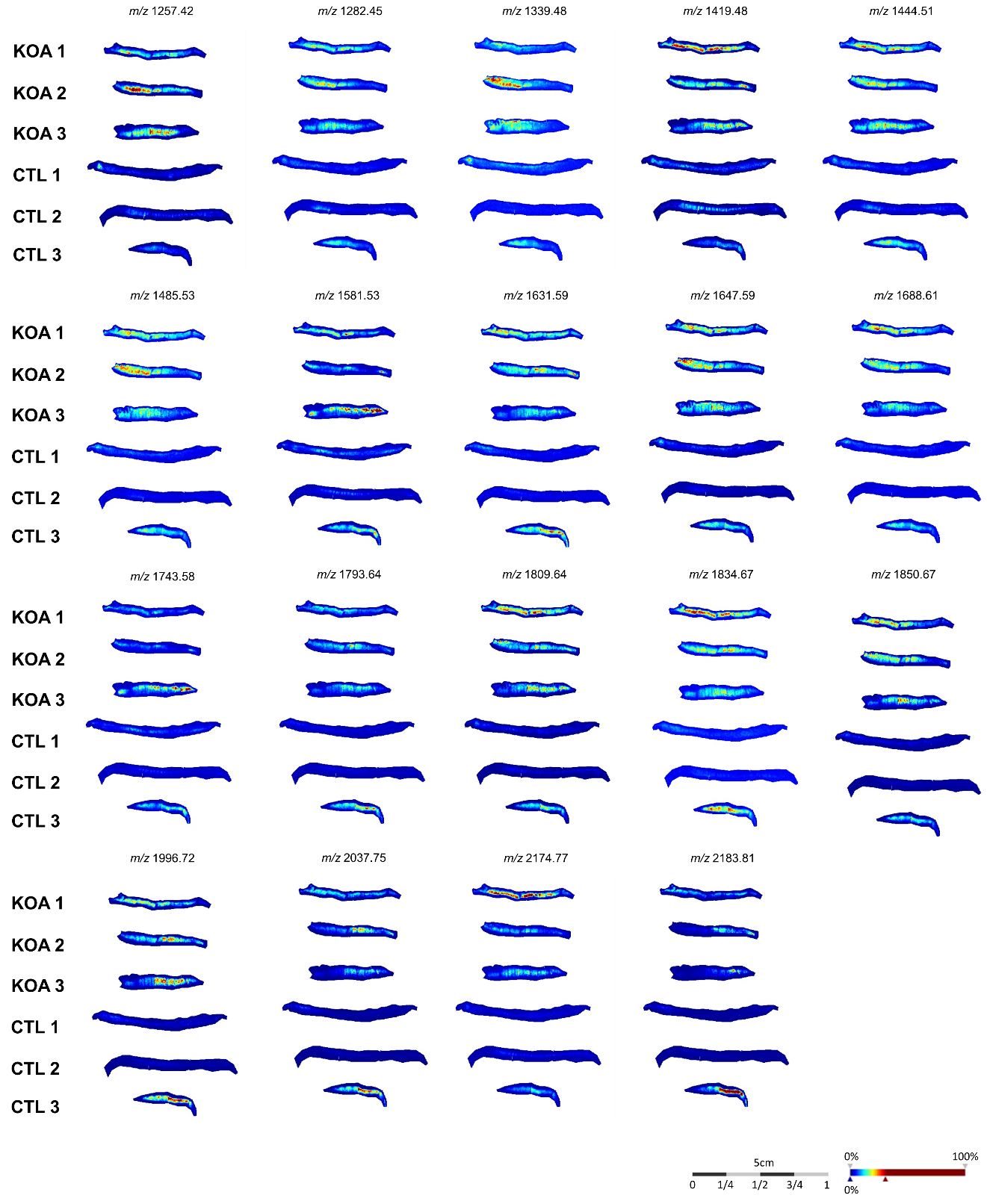


**Supplementary Figure 3.** *N*-glycan MALDI-MSI of FFPE cartilage tissue from CTL individuals (n=3) and KOA patients (n=3). Ion intensity maps were generated and visualized for individual masses ± 0.3 Da using SCiLS Lab software.
